# Development of Cross-Reactive Live Attenuated Influenza Vaccine Candidates against Both Lineages of Influenza B Virus

**DOI:** 10.3390/vaccines12010095

**Published:** 2024-01-18

**Authors:** Pei-Fong Wong, Irina Isakova-Sivak, Ekaterina Stepanova, Elena Krutikova, Ekaterina Bazhenova, Andrey Rekstin, Larisa Rudenko

**Affiliations:** Department of Virology, Institute of Experimental Medicine, 197022 St. Petersburg, Russia; po333222@gmail.com (P.-F.W.); stepanova.ea@iemspb.ru (E.S.); krutikova.iem@mail.ru (E.K.); sonya.01.08@mail.ru (E.B.); arekstin@yandex.ru (A.R.); vaccine@mail.ru (L.R.)

**Keywords:** influenza B virus, live attenuated influenza vaccine, reverse genetics, recombinant influenza virus, cross-protection, viral immunity

## Abstract

Background: Influenza viruses continue to cause a significant social and economic burden globally. Vaccination is recognized as the most effective measure to control influenza. Live attenuated influenza vaccines (LAIVs) are an effective means of preventing influenza, especially among children. A reverse genetics (RG) system is required to rapidly update the antigenic composition of vaccines, as well as to design LAIVs with a broader spectrum of protection. Such a system has been developed for the Russian LAIVs only for type A strains, but not for influenza B viruses (IBV). Methods: All genes of the B/USSR/60/69 master donor virus (B60) were cloned into RG plasmids, and the engineered B60, as well as a panel of IBV LAIV reassortants were rescued from plasmid DNAs encoding all viral genes. The engineered viruses were evaluated in vitro and in a mouse model. Results: The B60 RG system was successfully developed, which made it possible to rescue LAIV reassortants with the desired antigenic composition, including hybrid strains with hemagglutinin and neuraminidase genes belonging to the viruses from different IBV lineages. The LAIV candidate carrying the HA of the B/Victoria-lineage virus and NA from the B/Yamagata-lineage virus demonstrated optimal characteristics in terms of safety, immunogenicity and cross-protection, prompting its further assessment as a broadly protective component of trivalent LAIV. Conclusions: The new RG system for B60 MDV allowed the rapid generation of type B LAIV reassortants with desired genome compositions. The generation of hybrid LAIV reassortants with HA and NA genes belonging to the opposite IBV lineages is a promising approach for the development of IBV vaccines with broad cross-protection.

## 1. Introduction

Licensed seasonal influenza vaccines are available globally as inactivated influenza vaccines (IIV), recombinant influenza vaccines (rIV) or live attenuated influenza virus (LAIV) vaccines [1]. LAIVs are produced using master donor viruses (MDVs), which carry a series of mutations that restrict virus replication to the upper respiratory tract [2]. LAIVs based on the A/Ann Arbor/6/60 (H2N2) and B/Ann Arbor/1/66 viruses were developed in the USA [3,4,5]. In Russia, LAIVs based on A/Leningrad/134/17/57 (A/Len/17) and B/USSR/60/69 (B60) viruses have been used since the 1960s [6,7,8]. Traditional LAIVs contain the surface antigens hemagglutinin (HA) and neuraminidase (NA) of seasonal influenza viruses recommended by the World Health Organization (WHO) for a particular season, and the remaining six viral segments of an attenuated master donor virus (MDV) [9]. At present, only traditional reassortment has been used to generate candidate vaccine viruses to produce IBV in Russia [10]. However, reassortment does not allow the targeted engineering of viral genes, limiting the scope of investigations, and takes a great deal of time [11]. Plasmid-based reverse genetics techniques (RG) provide an alternative method to generate IIV, rIV and LAIV [12]. Currently in Russia, LAIV reassortant strains for IAVs based on A/Leningrad/134/17/57 (H2N2) MDV can be quickly generated using reverse genetics systems [13]. Although live attenuated influenza B vaccine strains based on MDV B60 can be produced by classical reassortment, the need for plasmid-based reverse genetics system of MDV B60 remains.

Isolated in 1940 [14], influenza type B virus (IBV) belongs to the *Orthomyxoviridae* family and consists of eight segments of linear negative-sense, single-stranded RNA. Since the 1970s, IBVs have diverged into two antigenically distinct virus lineages—B/Yamagata (represented by B/Yamagata/16/88) and B/Victoria (represented by B/Victoria/2/87) lineages [15,16,17]. The two lineages are antigenically distinct, so little or no cross-neutralizing antibody is observed after infection [18]. Although IBV generally causes milder disease than IAV and primarily affects children [19,20], adults and the elderly can also be affected and severe illness requiring hospitalization is still frequently observed [21]. IBV can cause seasonal epidemics and can be transferred throughout the year [22]. In the case of trivalent influenza vaccine formulation, inaccurate prediction of the predominant IBV lineage leaves many vaccinated individuals with suboptimal protection against IBV infection caused by the IBV lineage not included in the licensed trivalent vaccine [23]. The recently developed quadrivalent vaccine protects against both B lineages [24]. The HA genes of the B/Victoria and B/Yamagata viruses evolve at a slower pace than in IAV lineages, although the Victoria lineage undergoes faster rates (average of 3.9–5.1 years) compared to the Yamagata lineage (average of 6.3–7.2 years) [25]. Yamagata viruses diverged before the year 2000, and two antigenically distinct clades cocirculated exhibiting an epidemiological pattern of alternating antigenic dominance between seasons. Since 2011, the Victoria lineage has also diversified into two dominant monophyletic clades. To minimize the impact of B-mismatch on vaccine effectiveness, in 2013, the WHO recommendations included a second influenza B strain in the vaccine composition and later two QIVs have been developed: a live-attenuated QIV and a split inactivated QIV [26]. However, vaccine effectiveness may still be reduced in the case of antigenic drift within either IBV lineage [25]. In addition, for LAIVs, a reduction in vaccine effectiveness has been noted when the fourth component was added to the trivalent composition, most probably due to the interference between the IBV and IAV strains [27]. Therefore, the development of a cross-lineage IBV vaccine, effective to generate cross-reactive antibodies against both B/Yamagata and B/Victoria lineages, would be valuable in providing broad protection.

In this study, we developed an eight-plasmid-based RG system for the MDV B60 and sought to evaluate the phenotypic and immunological properties of the LAIV strains generated by reverse genetics compared with the IBV vaccine candidates produced by classical reassortment in eggs. In addition, new cross-lineage live attenuated IBV vaccine prototype reassortants with a 6 + 1 + 1 genome composition on the B60 backbone were developed and assessed in a mouse model of IBV infection.

## 2. Materials and Methods

### 2.1. Cell Lines and Virus Strains

Vero cells purchased from ATCC were maintained in OptiPRO SFM medium supplemented with 1% antibiotic/antimycotic solution (both from Gibco, Grand Island, NY, USA) without fetal bovine serum (FBS) (Capricorn, Düsseldorf, Germany). Madin-Darby canine kidney (MDCK) cells and Hek293T (293T) cells purchased from ATCC were maintained in Dulbecco’s modified Eagle’s medium (DMEM) (Gibco, USA) supplemented with 10% FBS and 1% antibiotic/antimycotic solution (Sigma-Aldrich, Saint Louis, MO, USA). Cells were propagated at 37 °C in a humidified incubator under a 5% CO_2_ atmosphere.

The B/Victoria/2/87-like (B/Vic) B/Brisbane/60/2008 (Br-wt) and B/Yamagata/16/88-like (B/Yam) B/Phuket/3037/13 (Ph-wt) strains were provided by the CDC (Atlanta, GA, USA). Cold-adapted master donor virus (MDV) B/USSR/60/69 (B60) and classical LAIV reassortants B/60/Brisbane/60/2008 vaccine (Br-vac), and B/60/Phuket/3037/2013 (Ph-vac), as well as the wild-type B/Lee/40 IBV, were obtained from the influenza virus repository of the Department of Virology of the Institute of Experimental Medicine (Saint Petersburg, Russia). A mouse-adapted B/Malaysia/2506/2004 (Ma-wt) virus was obtained from the Smorodintsev Research Institute of Influenza (Saint Petersburg, Russia). Virus stocks were amplified in embryonated chicken eggs (Poultry farm “Sinyavinskaya”, Leningrad region, Russia), and were stored at −70 °C in single-use aliquots. 

### 2.2. Plasmids Cloning

The reverse genetics system for the IBVs strain has been described previously [28]. Primers for RT-PCR were designed based on the consensus sequences of each IBV gene’s termini and for recognition sequences for the restriction endonuclease SapI generation sticky ends. The primers used for the construction of plasmids representing the eight segments of the B60 and the HA and NA genes of B/Brisbane/60/2008 and B/Phuket/3037/2013 are shown in Table S1. The QIAamp Viral RNA Kit (Qiagen, Germantown, MD, USA) was used to extract viral RNA from 100 µL of allantoic fluid from infected embryonated chicken eggs. For RT-PCR of the eight segments of RNA of IBVs, the One Step RT-PCR kit (Invitrogen, Carlsbad, CA, USA) was used according to the protocol provided. The bidirectional cloning vector pCIPolISapIT was previously described [29]. The cloning vector allows the insertion of sequences of interest between the pol I promoter and the terminator using the SapI endonuclease. The pol I transcription unit is flanked by the pol II promoter from cytomegalovirus and the late mRNA polyadenylation signal from the SV40 virus. The plasmid contains a 430-nucleotide spacer sequence between two XbaI restriction sites that can be used to scan the insert. Viral RNAs were isolated using the QIAamp Viral RNA Kit (Qiagen, USA). The PB2, PB1, HA, NA, M and NS genes of the B60 strain were cloned by hydrolysis of the DNA coding fragments after PCR with SapI endonuclease, then the hydrolyzed fragments were ligated into the SapI restriction site of the pCIPolISapIT vector using T4 ligase (Invitrogen, USA). The PA gene of the B60 strain was cloned from two parts of gene fragments digested with BsmBI endonuclease and triple ligated to the pPol12BB vector. The NP gene was cloned by overlapping PCR, which was templated by fragments of two private genes from the first round of amplification. After overlapping PCR, the NP coding fragments were digested with SapI restriction endonucleases and ligated to the pCIPolISapIT vector. 

Two chimeric NA (cNA) plasmids were constructed through PCR amplification with specific primers (Table S1) to replace untranslated regions (UTR) of type B NA to appropriate regions of A/Len/17 NA. 

Competent cells of the *E. coli* strain XL10-Gold (Agilent technologies, Santa Clara, CA, USA) were transformed with ligation mixtures. Transformant colonies were scanned for inserts of the correct length by hydrolysis with XbaI endonuclease. Extracted plasmid DNAs were subjected to Sanger sequencing, and in cases where the plasmids had mutations resulting in amino acid changes, they were corrected to reflect the consensus sequence by site-directed mutagenesis using the Site-Directed Mutagenesis kit (Invitrogen, USA).

### 2.3. Influenza Virus Rescue

Two approaches were used to reconstitute infectious influenza viruses from plasmid DNAs: electroporation of Vero cells or lipofection of co-cultured 293T/MDCK cells.

For electroporation, 2 µg of each plasmid DNA was combined into a 1.5 mL tube for each reaction, mixed with NaOAc buffer and incubated overnight at −20 °C. The plasmid set used for transfection is shown in Table 1. The next day, plasmid DNAs were precipitated by centrifugation, washed with 70% ethanol, and resuspended in 8 µL of TE buffer. Vero cells were harvested and diluted to 5 × 10^6^ cells/mL for each reaction in OptiPRO medium supplemented with 1× Glutamax, 1× antibiotic-antimycotic (Gibco, Grand Island, NY, USA), and then were transfected using the Neon™ transfection system (Thermo Fisher Scientific, Carlsbad, CA, USA) according to the manufacturer’s instructions. After 6 h incubation at 37 °C, the transfection medium was replaced with 2 mL of OptiPRO containing 1× Glutamax and 2.5 μg/mL trypsin (Sigma) and incubated at 33 °C for 3–7 days. Then, the tissue culture supernatant was used to inoculate 10–12-day-old eggs, followed by incubation at 33 °C for 3 days. The presence of infectious virus was determined by hemagglutination (HA) assay with 0.5% chicken red blood cells. Working viral stocks were prepared by an additional passage of the RG virus in eggs. 

One day before lipofection, a mixture of 2 × 10^5^ MDCK and 4 × 10^5^ HEK293T cells was seeded on a 6-well plate in 3 mL of DMEM (Dulbecco’s modified Eagle medium) supplemented with 1× antibiotic-antimycotic and 10% FBS (Capricorn, Germany). The next day, the medium was replaced with 1 mL of DMEM supplemented with 5% FBS, but without antibiotics. For each transfection sample, 1 µg of each plasmid DNA was diluted in 50 μL of DMEM without serum. In a separate tube, 16 μL of GenJect-39 reagent (Molecta, Moscow, Russia) was diluted in 50 μL of DMEM and incubated for 10 min at room temperature (RT). After the incubation, the diluted DNA was mixed with diluted GenJect-39 and incubated for 20 min at RT. This mixture was further added dropwise to each well containing cells and medium. The cells were incubated at 37 °C in a CO_2_ incubator overnight. After 16 h of incubation, the wells were washed once with 1 mL PBS to remove FBS, and then 3 mL of DMEM containing 1× antibiotic-antimycotic and 2.5 μg/mL trypsin (Sigma) was added to each well, followed by a 3-day incubation at 33 °C. Viral stocks were then generated as described above.

### 2.4. Assessment of Virus Growth Characteristics

#### 2.4.1. Replication in Eggs

To evaluate virus replication activity in eggs (EID_50_), virus stocks were 10-fold-serially diluted in PBS and inoculated into a 10–12-day-old embryonated chicken egg in a volume of 200 µL per egg. After 3 days of incubation at 33 °C, the chorio-allantoic fluid was tested for HA activity and the infectious virus titers were calculated according to the method of Reed and Muench [30].

Temperature-sensitive and cold-adapted phenotypes were also assessed in eggs. These phenotypes were estimated by side-by-side titration of the influenza B viruses in 10-day-old eggs at permissive (33 °C) and non-permissive low (26 °C) and high (37 and 38 °C) temperatures. Eggs were infected as described above and incubated for 3 days at 33, 37 or 38 °C or 6 days at 26 °C. The virus was considered as temperature-sensitive if the difference between the virus titer at 33 °C and 37–38 °C was not less than 5.0 logEID_50_. The virus was considered as cold-adapted if the difference between the virus titer at 33 °C and 26 °C was less than or equal to 3.0 logEID_50_.

#### 2.4.2. Replication in MDCK Cells

To evaluate the fifty-percent tissue culture infective dose (TCID_50_) in MDCK cells, virus stocks were 10-fold-serially diluted in infection medium (DMEM, supplemented with 1× antibiotic-antimycotic solution and 1 µg/mL TPCK trypsin) and added to the 96-well plates with a confluent cell monolayer thoroughly washed with warm PBS, in a volume of 25 µL per well. After 1 h of adsorption at 33 °C, the inoculum was removed and the cells were washed with PBS, and 150 µL of infection medium was added to each well, followed by 72 h incubation at 33 °C, 5% CO_2_. The supernatants were then tested for HA activity, and the infectious virus titers were calculated according to the method of Reed and Muench [30]. 

The growth kinetics of the reassortant viruses were compared at 33 °C. Confluent monolayers of MDCK cells grown on 6-well plates were inoculated in triplicate at a multiplicity of infection (MOI) of 0.001 for each virus. At 0, 1, 2, 3 and 4 days post inoculation (dpi), culture supernatants were collected and stored at −70 °C for virus titer quantification. Virus titers were determined by TCID_50_ assays as described above.

### 2.5. Virus Concentration and Purification

Embryonated chicken eggs were inoculated with influenza viruses at a dose of 4–5 logEID_50_. After 3 days of incubation at 33 °C, the chorio-allantoic fluid from each egg was collected and pooled. The infectious culture fluid was clarified by low-speed centrifugation, followed by ultracentrifugation at 19,000 rpm for 2.5 h at 4 °C using an Optima L-100 XP centrifuge (Beckman Coulter, Brea, CA, USA). After removing the supernatant, the pellet was re-suspended in 1 mL of cold PBS. These samples were then loaded onto a discontinuous gradient (sequentially layering 60 and 30% sucrose solution (5 mL + 5 mL) into 14 mL centrifuge tubes) and centrifuged for 90 min at 23,000 rpm at 4 °C. After ultracentrifugation, the white band between the sucrose layers was extracted and retained. The virus band was transferred to 14 mL centrifuge tubes, diluted with PBS and centrifuged for 1 h at 23,000 rpm at 4 °C. Sediments were dissolved in PBS and stored at −70 °C in single-use aliquots.

### 2.6. Animal Experiments

This study includes several independent experiments involving 6–8-week-old female C57BL/6J mice purchased from the animal farm Stolbovaya (Moscow Region). All experimental studies, procedures and manipulations on animals were carried out by the staff who have many years of experience in involving various experimental animals as an object of study. The study was approved by the Local Ethics Committee of the Institute of Experimental Medicine (ethical approval number #1/20 dated 27 February 2020 and #1/23 dated 20 April 2023).

Groups of mice were inoculated intranasally with 6.0 logEID_50_ of the studied vaccine viruses twice, at a 3-week interval. On Day 3 post-inoculation, 4 animals from each vaccine group were humanely euthanized for tissue collection. Nasal turbinates (NT) and lungs were collected for virus titer quantification. On Day 43 of the study, 8 mice from each group were bled via the retroorbital sinus to obtain serum samples for measuring influenza-specific antibody responses. At 46 dpi, each group was subjected to challenge infections with different wild-type IBVs. For some experiments, respiratory tissues were collected on Days 3 (*n* = 4) and 6 (*n* = 4) after challenge to determine viral titers; in other experiments, challenged animals (*n* = 5) were monitored for survival and weight loss for 14 days post-challenge, and were scored as dead and humanely euthanized if they lost more than 30% of their initial body weight.

To evaluate the fifty-percent mouse lethal dose (MLD_50_) of the challenge viruses, serial viral dilutions were inoculated intranasally in 50 µL to groups of mice (*n* = 3–4). Animals were monitored for survival and weight loss for 14 days post-challenge. The MLD_50_ value was calculated using the Reed and Muench method.

The indirect protective effect of antibody in collected serum specimens was assessed in mouse in vivo protection experiments as described elsewhere [31]. Serum samples from each study group were pooled in equal volumes from each mouse and this mixture was combined with PBS at a 1:1 ratio, followed by heat-inactivation at 56 °C for 1 h. Then, 3 MLD_50_ of one of the WT IBVs was added to each treated sample at equal volumes and these mixtures were incubated for 30 min at room temperature. Finally, naïve C57BL/6J mice were i.n. infected with the prepared virus-serum mixtures, and the protective effect of sera was assessed by monitoring mouse survival and weight loss for 14 days post-infection. Mice were scored as dead and humanely euthanized if they lost more than 30% of their initial body weight.

### 2.7. Immunological Methods

Serum samples collected from immunized mice were assessed for the presence of influenza virus-specific antibodies using hemagglutination inhibition (HAI) assay, ELISA, microneutralization (MN) assay or neuraminidase inhibition assay (NAI).

#### 2.7.1. Hemagglutination Inhibition Assay

Serum samples collected at 43 dpi were assayed for the presence of HAI antibodies as described elsewhere [32]. The B/Br-wt and B/Ph-wt viruses were used as antigens to test serum samples in this study. Serum samples were treated with a receptor destroying enzyme (RDE) (DENKA SEIKEN Co., Ltd., Tokyo, Japan) for 18–20 h at 37 °C, followed by heat-inactivation at 56 °C for 1 h, following by the addition of PBS to the mixture to yield a final serum dilution of 1:10. Two-fold serial dilutions of serum samples (1:10–1:1280) were made in 96-well U-bottom microtiter plates. An equal volume of PBS containing 4 hemagglutinating units of the virus was added to each well and incubated for 1 h at RT. Finally, 0.5% chicken RBCs were added to the plate and incubated at RT for 30 min, followed by reading the hemagglutination patterns. The HI titer was defined as the reciprocal of the last serum dilution that completely inhibited hemagglutination, and titers < 10 were assigned a value of 5 for calculation purposes.

#### 2.7.2. ELISA

Serum samples collected after two immunizations were assayed for the presence of IgG antibodies by standard ELISA. Briefly, high-sorbent ELISA plates (Greiner bio-one, Frickenhausen, Germany) were coated with sucrose-gradient-purified IBVs at a concentration of 16 HA units per well, in 50 μL of coating buffer (0.5 M sodium bicarbonate buffer, pH 9.6). After overnight incubation at 4 °C, the plates were washed 2 times with PBST (PBS with 0.05% Tween 20) and blocked with 50 μL of 1% (*w*/*v*) bovine serum albumin (BSA) in PBS at 37 °C. Half an hour later, plates were washed with PBST 2 times, and 50 μL of serially diluted sera (1:20 to 1:20,480) was added to the plates and incubated at 37 °C for 1 h. Plates were washed and a 1:5000 dilution of horseradish peroxidase (HRP)-conjugated goat anti-mouse IgG (BioRad, Hercules, CA, USA) was then added to the plates, which were incubated for 30 min at 37 °C. After the final wash, 50 μL of 3,3′,5,5′-tetramethylbenzidine (TMB) (Thermo Scientific, Rockford, IL, USA) was added to the plates for 15 min. The reaction was stopped by adding 25 μL of 1M sulfuric acid (H_2_SO_4_) to each well. The plates were read at 450 nm by using a microplate spectrophotometer xMark (Bio-Rad, Irvine, CA, USA).

#### 2.7.3. Microneutralization Assay

The WT IBVs were used as antigens for the MN assay in this study. Samples were tested by MN in MDCK cells. Serum samples were treated with RDE for 18–20 h at 37 °C, followed by heat-inactivation at 56 °C for 1 h. Then, the dilutions were adjusted to 1:10 by the addition of PBS. Two-fold serial dilutions of sera were prepared in 96-well plates (1:20–1:2560) such that the final volume per well was 50 µL. Dilutions were performed in DMEM supplemented with a 1× solution of antibiotic-antimycotic and TPCK trypsin (1 µg/mL). Diluent and a positive serum against the WT IBVs were used as negative and positive controls, respectively. An equal volume of virus dilution containing 100 TCID_50_ per 50 µL was added to each well containing sera. Plates were incubated at 37 °C for 1 h. Following the incubation, 100 µL of virus + serum mixture was added into MDCK cells cultured in a 96-well format. Plates were incubated at 33 °C, 5% CO_2_ for 18–20 h. After incubation, the media was removed and the cells were washed with 200 µL of PBS, then pre-chilled (to −20 °C) 80% acetone diluted in PBS. The plates were incubated at 20 °C for 20 min, then the fixative was removed, and the plates were air dried. Cell-based ELISA was further performed to detect and quantify the expression of influenza B virus nucleoprotein (NP) in infected cells. For this, fixed cells were blocked with 5% non-fat milk diluted in PBST at room temperature for 30 min. Then, the blocking buffer was removed and the cells were quenched by the addition of 100 µL of 3% hydrogen peroxide (in PBS) and incubated for 20 min at room temperature. After washing three times with 200 µL PBST, peroxidase-conjugated anti-NP IBV monoclonal antibodies (LLC Enterprise for Production of Diagnostic Preparations, Saint Petersburg, Russia) diluted 1:4000 in PBST with 5% non-fat dry milk were added to each well. Plates were then incubated at room temperature for 1 h, followed by five-times washing with 200 µL PBST and the addition of TMB peroxidase substrate. The reaction was stopped by adding 50 µL of the 2M of sulfuric acid to all wells. Optical density at 450 nm was read by a microplate spectrophotometer (Bio-Rad). The reciprocal serum dilution corresponding to the highest dilution with OD_450_ less than 50% of the cut-off (≥50% inhibition) was considered the neutralization antibody titer for that serum sample.

#### 2.7.4. Neuraminidase Inhibition Assay

The enzyme-linked lectin assay (ELLA) was used to measure NAI titers as described elsewhere [33]. The 7 + 1 chimeric influenza A viruses containing the HA of H2N2 IAV and the NAs from the corresponding IBVs were generated by RG and were purified on a sucrose gradient as described above. The NAs of these viruses express the ectodomain (stalk and head) of the IBVs (B/Brisbane/60/2008 or B/Phuket/3073/2013). Serum samples were pre-treated with RDE for 18–20 h at 37 °C, followed by heat-inactivation at 56 °C for 1 h and the subsequent addition of PBS to yield a final serum dilution of 1:10. Two-fold serial dilutions of sera were prepared in 96-well plates (1:10–1:5120) such that the final volume per well was 50 µL. Dilutions were performed using 1% BSA in PBS. An equal volume of virus dilution that gives approximately 90% of the maximum signal and is within the linear range was added to each well containing serum dilutions. Diluent was used as a negative control, while a virus-only sample was used as a positive control. Plates were incubated at 37 °C for 30 min. Then, 100 µL of virus + serum mixture was transferred to the corresponding wells of the plate coated with fetuin (50 µg/mL). Plates were incubated at 37 °C for 1 h, followed by six-times washing with 200 µL PBST and the addition of 100 µL of PNA-HRPO solution (Sigma, USA) at a concentration of 2.5 µg/mL with subsequent incubation at RT for 1 h. After washing five times with 200 µL PBST, 50 μL of TMB (Thermo Scientific, USA) was added to the plates for 15 min. The reaction was stopped by adding 25 μL of 1M sulfuric acid (H_2_SO_4_) to each well. The plates were read at 450 nm by using a microplate spectrophotometer (Bio-Rad, USA). The percent of NA inhibition at each serum dilution was calculated using the formula:

% NA inhibition = 100 − 100 × (OD_sample_ − OD_NC_)/(OD_VC_ − OD_NC_)

where NC is the negative control, and VC is the virus-only control (wells with virus without sera).

The 50% NAI titer was calculated using four-parametrical non-linear regression analysis and the GraphPad Prism 10.1 software.

### 2.8. Statistical Analyses

Statistical analysis was performed using GraphPad Prism 10.1. Statistically significant differences between study groups were determined by ANOVA with Tukey’s multiple comparison test. Survival rates after challenge were analyzed by a log-rank Mantel–Cox test. Differences with *p* ≤ 0.05 were considered significant.

## 3. Results

### 3.1. Cloning of B60 Genese into RG Plasmids and Rescue of Recombinant IBVs

The 5′ and 3′ termini of IBV RNA segments are short conserved complementary regions that form the promoter for the IBV RNA-dependent RNA polymerase. The 5′ and 3′ termini of vRNAs or cRNAs are different between influenza type A and type B viruses and contain sequences that are unique for each of the eight segments [28]. Primers were designed based on the consensus sequences of termini, with consideration of natural variations.

To construct eight plasmids representing the master donor virus B/USSR/60/69, viral RNA was reverse transcribed and amplified by PCR. The genes PB2, PB1, HA, NA, M and NS were cloned through hydrolysis by SapI endonuclease for amplified full-size copies of viral genes and ligated with SapI-digested vectors. The PA gene was cloned through the amplification of two fragments of the gene followed by hydrolysis with BsmBI endonuclease facilitating the exact fusion of the two amplified fragments in a four-fragment ligation reaction with SapI-digested vectors. The NP gene was cloned using the strategy of overlapping PCR, carried out using two previously amplified fragments of the NP gene. The resultant PCR fragments of the NP gene were fused via a second round of PCR followed by hydrolysis with SapI endonuclease and ligated with SapI-digested vectors. All constructed plasmids were verified via Sanger sequencing analysis and diagnostic restriction with the XbaI endonuclease (Figure S1A). Therefore, all viral cDNAs cloned into the RG plasmids were sequenced in their entirety and represent the consensus sequence of the eight segments of B/USSR/60/69. These plasmids were designated PB2-B60-pCIPolISapIT, PB1-B60-pCIPolISapIT, PA-B60-pCIPolISapIT, HA-B60-pCIPolISapIT, NP-B60-pCIPolISapIT, NA-B60-pCIPolISapIT, M-B60-pCIPolISapIT and NS-B60-pCIPolISapIT. There were no nucleotide differences between the sequences of the native virus and those of the plasmids used for subsequent transfections. 

For HA and NA cloning of WT viruses, the primers were optimized for simultaneous RT-PCR amplification of the HA and NA segments. Both HA and NA gene fragments of B60 MDV and B/Brisbane/60/2008 and B/Phuket/3037/2013 were successfully cloned into the RG vector. Four plasmids, HA-B/Brisbane-pCIPolISapIT, NA-B/Brisbane-pCIPolISapIT, HA-B/Phuket-pCIPolISapIT and NA-B/Phuket-pCIPolISapIT, were constructed and verified via Sanger sequencing analysis and diagnostic restriction digest (XbaI) (Figure S1B).

To test whether infectious the B/USSR/60/69 virus could be reconstituted from plasmid DNAs, Vero cells were transfected with eight plasmids carrying all genes of B/USSR/60/69. Characteristic virus-induced cytopathic effect (CPE) was evident in the Vero cells 3 to 4 days post transfection. The mixture of transfection culture and cells was inoculated into eggs, and the viable virus was detected after 3 days of incubation at 33 °C. The virus presence in allantoic fluid from each egg was verified by hemagglutination assay, and the HA titer at passage E2 was found to be 1:1024. These results show that the B60-RG virus could be efficiently and reliably generated from the eight plasmids and that this recombinant virus has the same high-yield phenotype as the prototype virus.

To demonstrate the utility of the developed B60 RG system for the efficient generation of LAIV reassortants, two recombinant vaccine reassortant viruses were generated by the transfection of Vero cells by a mixture of eight plasmids containing PB1, PB2, PA, NP, M and NS from B60, and the HA and NA from strains representing both the Victoria and Yamagata lineages (Table 1). The B/Victoria lineage was represented by the B/Brisbane/60/2008 virus, whereas B/Yamagata lineage was represented by the B/Phuket/3037/2013 strain. Both LAIV RG reassortants were successfully rescued and the HA titers of the egg-grown viruses were 1:128. Sanger sequencing confirmed the identity of all viral genes as those of the prototype viruses.

### 3.2. Comparing Replicative Activity of Engineered IBVs and Their Classical Counterparts 

The replicative characteristics of the non-RG, RG and WT viruses were studied in eggs incubated at different temperatures. The B/USSR/60/69 MDV has a temperature-sensitive (*ts*) cold-adapted (*ca*) phenotype, which is primarily conferred by the viral internal proteins and inherited by the 6 + 2 LAIV reassortants [34]. Nevertheless, at 37 °C, both wild-type viruses had a *ts* phenotype even more pronounced than the B60 strain (Table 2). For recently circulating IBVs, restricted growth at 38 and 37 °C is common [35], and this phenotypical marker could not be used for distinguishing between the WT and LAIV strains. At 26 °C, all tested viruses, except Br-wt, were cold-adapted. In general, B60-RG and both rescued LAIV reassortant viruses were similar to their classical counterparts in terms of growth characteristics in eggs at different temperatures.

**Figure 1:**
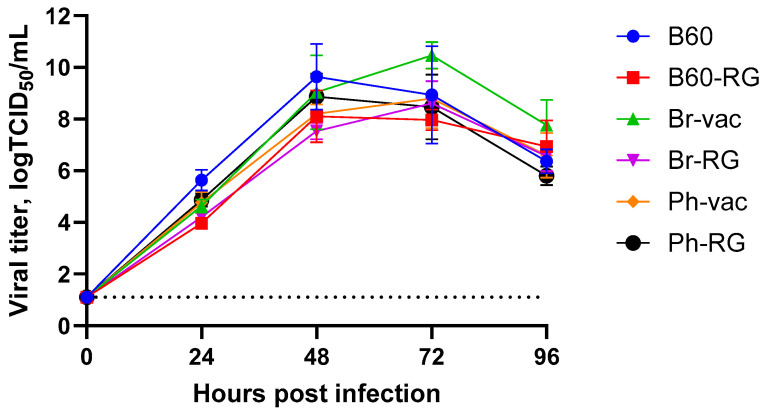
Growth characteristics of recombinant LAIV viruses in MDCK cells. Confluent monolayers of MDCK cells were inoculated at an MOI of 0.001 with either the MDV or 6 + 2 (RG and non-RG) and incubated at 33 °C. Culture supernatants were collected at 0, 24, 48, 72 and 96 hpi, and viral titers were quantified by TCID_50_ assays. Dotted line represents the limit of virus detection in the TCID_50_ assay.

Furthermore, both B60 and B60-RG, as well as the two engineered LAIV reassortants and their classical counterparts, grew efficiently in MDCK cells. Their titers ranged from 7.8 to 8.5 logTCID_50_/mL (Table 2), and the growth kinetics on cells infected with MOI = 0.001 were rather similar, reaching peak titers of no less than 8.0 logTCID_50_/mL, suggesting the feasibility of LAIV production using cell culture technologies (Figure 1).

**Figure 2:**
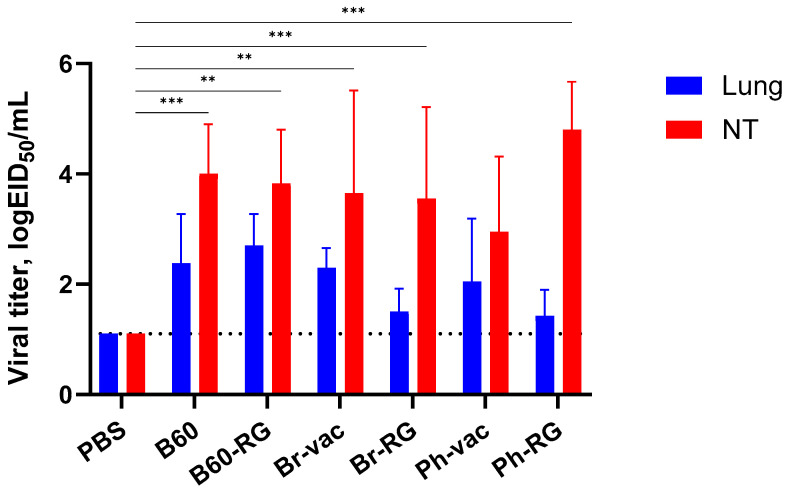
Replication of the studied influenza B viruses in mouse respiratory tissues. Viruses were administered i.n. to groups of mice (*n* = 4) at a dose of 10^6^ EID_50_. Lungs and nasal turbinates (NT) were collected on Day 3 p.i. and viral titers were determined by the titration of tissue homogenates in eggs. Data were compared using two-way ANOVA with Tukey’s post-hoc multiple analyses test. ** *p* < 0.01, *** *p* < 0.001. Dotted line represents the limit of virus detection in the EID_50_ assay.

Next, we assessed the ability of the RG and non-RG IBVs to replicate in the upper and lower respiratory tract of C57BL/6J mice after i.n. inoculation of 10^6^ EID^50^ of each virus. As expected, all viruses replicated efficiently in nasal turbinates, whereas their replication was less pronounced in the lungs, suggesting the attenuated phenotypes of all studied viruses (Figure 2). No significant differences were observed for RG viruses compared to the corresponding non-RG variant, thus indicating the preservation of the main replicative properties of type B LAIV reassortants generated from plasmid DNAs.

### 3.3. Comparing Immunogenicity and Protective Activity of Engineered IBVs and Their Classical Counterparts in a Mouse Model

**Figure 3:**
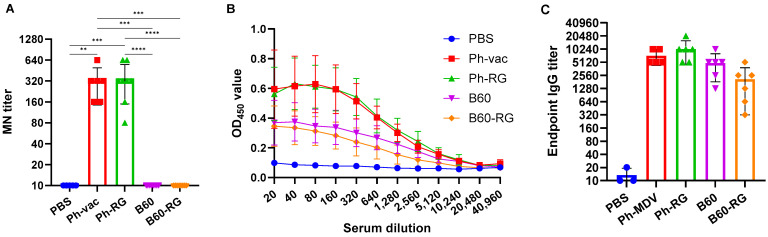
Antibody immune responses to the RG and non-RG influenza B viruses in mice after two immunizations. (**A**) Neutralizing antibody titers to the Ph-wt virus as measured by MN assay. (**B**) Binding levels of serum IgG antibodies to the purified Ph-wt whole virus as measured by ELISA. (**C**) Endpoint serum IgG antibody titers to the Ph-wt virus determined by ELISA. Data were analyzed by one-way ANOVA with Tukey’s post-hoc multiple analyses test. **—*p* < 0.01; ***—*p* < 0.001; ****—*p* < 0.0001.

For comparative assessment of immunogenic and protective properties of RG and non-RG viruses in mice, pairs of B60 versus B60-RG and Ph-vac versus Ph-RG viruses were selected. Mice were immunized i.n. at a dose of 6 logEID_50_, twice with a 3-week interval. In order to assess the antibody response after boosting, all mice were bled after 3 weeks before challenge, and MN assays were then performed against the homologous virus Ph-wt. All serum samples from Ph-RG and non-RG Ph-vac mice after primed-boost vaccination had high levels of MN antibody against the Ph-wt, suggesting an adequate and potentially protective antibody response according to current standards for surrogates of protection (Figure 3A). ELISAs were also performed to determine the IgG antibody levels against the Ph-wt. The binding signals of serum samples from both the Ph-RG and Ph-vac groups toward the homologous virus were comparable (Figure 3B). Although sera from animals that received Ph-based vaccine strains had binding signals significantly greater than responses in the B60 MDV groups of animals, the endpoint IgG antibody titers were not significantly different between study groups (Figure 3C). These data suggest that immunization with attenuated IBV viruses induces binding antibodies not only to the globular head domain of the virus, but also to other antigenic determinants that are similar between the LAIV reassortant and the B60 MDV.

**Figure 4:**
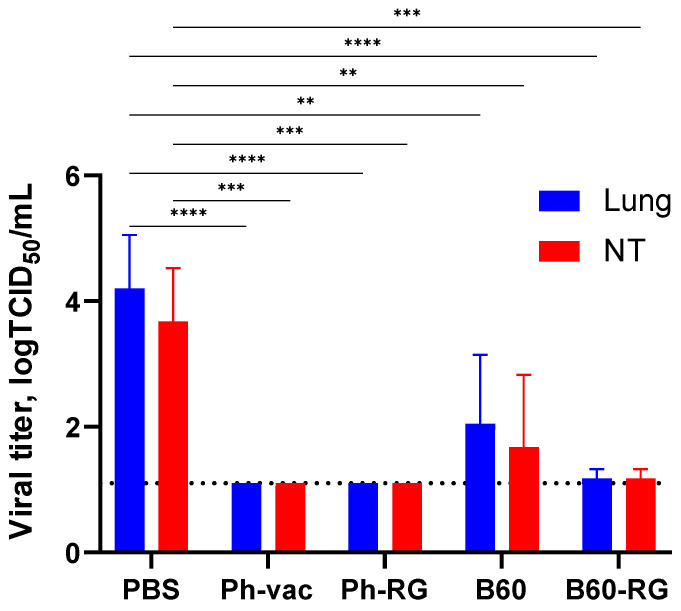
Protective activity of RG and non-RG influenza B viruses in mice. Immunized mice were challenged with B/Phuket/3037/2013 virus, and viral titers were determined in lungs and nasal turbinates on Day 3 post challenge. Data were analyzed by two-way ANOVA with Tukey’s post-hoc multiple analyses test. **—*p* < 0.01; ***—*p* < 0.001; ****—*p* < 0.0001. Dotted line represents the limit of virus detection in the TCID_50_ assay.

To assess protective activity of the studied viruses, immunized mice were challenged with Ph-wt virus. Lungs and NTs were collected on Day 3 p.c. (*n* = 4), and infectious virus titers in tissue homogenates were determined by TCID_50_ assay on MDCK cells. Strikingly, almost no infectious virus was observed in all vaccine groups. In contrast, the mock-immunized group showed comparably high lung and NT virus titers (Figure 4). These findings supported that both RG and non-RG vaccines protect mice from Ph-wt, accompanied by viral clearance in the lungs and NT. The observed protection of B60 MDV against heterologous infection can be explained by the action of cross-reactive T-cell responses; however, more experiments will be needed to confirm this assumption.

Overall, the RG constructed vaccines led to the induction of broadly reactive antibodies and were similar to their naturally derived counterparts produced by classical reassortment in terms of safety, immunogenicity and protective activity.

### 3.4. Generation of Hybrid IBV LAIV Candidates and Their Assessment In Vitro and In Vivo 

**Figure 5:**
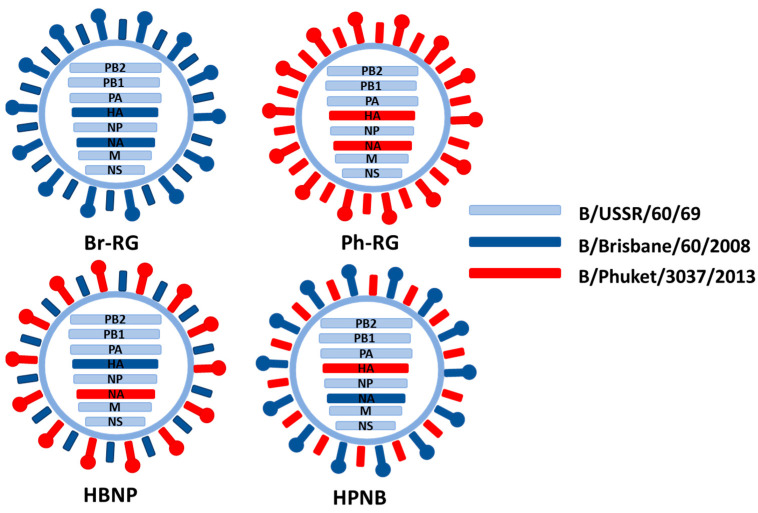
Hybrid (6 + 1 + 1) and control (6 + 2) recombinant viruses rescued for this experiment.

The developed RG system for B60 master donor virus was further explored to generate hybrid LAIV reassortants, as an attempt to design a vaccine protective against both IBV lineages. Two recombinant 6 + 1 + 1 hybrid LAIV strain with HA of Br-wt and NA of Ph-wt (named HBNP) or with HA of Ph-wt and NA of Br-wt (named HPNB) were generated (Table 1, Figure 5). For generation of 6 + 1 + 1 reassortants, co-cultured HEK239T and MDCK cells were used to rescue the virus. Both 6 + 1 + 1 reassortant viruses had titers between 8 and 9 logEID_50_/mL with an HA titer of 1:256 at E2 passage.

Replicative characteristics of the 6 + 1 + 1 recombinant viruses, along with corresponding 6 + 2 RG LAIV viruses were studied in eggs incubated at different temperatures. All rescued vaccine viruses had ts/ca phenotypes similar to the B/USSR/60/69 MDV (Table 3). Therefore, modification of the classical LAIV viruses by combining HA and NA genes from different IBV lineages did not have any significant consequences on the phenotypical properties of the vaccine viruses.

**Figure 6:**
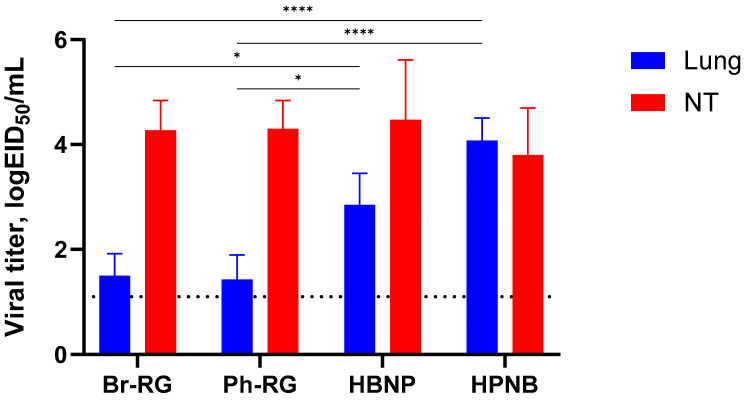
Safety and immunogenicity of viruses. Virus replication and tissue tropism of the 6 + 2 or 6 + 1 + 1 viruses in the respiratory tracts of mice. At 3 dpi, four animals from each group were euthanized, and virus titers in the upper respiratory tracts (nasal turbinates) or lower respiratory tracts (lungs) of the mice were determined by limiting dilutions in eggs. Data were analyzed by two-way ANOVA with Tukey’s post-hoc multiple analyses test. *—*p* < 0.05; ****—*p* < 0.0001. Dotted line represents the limit of virus detection in the EID_50_ assay.

To investigate whether the HA/NA replacement between two lineages of IBV would result in the attenuation of recombinant viruses in vivo, we evaluated its safety in mice. C57BL/6J mice were i.n. immunized with 10^6^ EID_50_ of each virus, and viral titers in lungs and NTs were determined on Day 3 p.i. Most of the viruses replicated well in the upper respiratory tract, as evidenced by virus detection in nasal turbinates (Figure 6). Strikingly, mice inoculated with the HPNB virus had levels of virus replication in the lower respiratory tract comparable to those of the NTs, suggesting the less attenuated phenotype of this virus compared to standard LAIV 6 + 2 LAIV reassortants. The HBNP virus also had slightly higher titers in the mouse lungs, but these titers were no more than 3 logEID_50_, and the viral replication was still more active in the NTs, which is an attribute of the attenuated influenza viruses (Figure 6). These data suggest that reassorting HA and NA genes between B/Victoria and B/Yamagata lineages may increase virus replication in the mouse respiratory tract, and the precise mechanism of such an effect remains to be elucidated. 

### 3.5. Immunogenicity of Hybrid IBV LAIV Candidates for C57BL/6J Mice

We further assessed the immunogenicity of the newly designed hybrid LAIV viruses by i.n. immunization of C57BL/6J mice with two doses of the 6 + 1 + 1 LAIV candidates, 10^6^ EID_50_ each, at a 3-week interval. Standard 6 + 2 LAIV reassortants (Br-RG and Ph-RG) were used as control vaccine viruses. Serum samples were collected 21 days after the second dose, and serum IgG antibody levels were measured by ELISA and HAI assay against the Br-wt and Ph-wt whole viruses. All serum samples from mice immunized with Ph-RG and HPNB had HAI antibody titers against the Ph-wt, suggesting an adequate and potentially protective antibody response according to current standards for surrogates of protection. As expected, HAI antibody titers against the Br-wt could be detected in mouse sera only from the groups Br-RG and HBNP, suggesting that Ph-wt and Br-wt are antigenically different from each other. Although HI antibody levels in the HBNP group were lower compared with the control group Br-RG, this difference was not statistically significant, suggesting that the antibody response to Br-wt was maintained when the NA gene in the Br-RG LAIV virus was replaced with that of Ph-wt.

**Figure 7:**
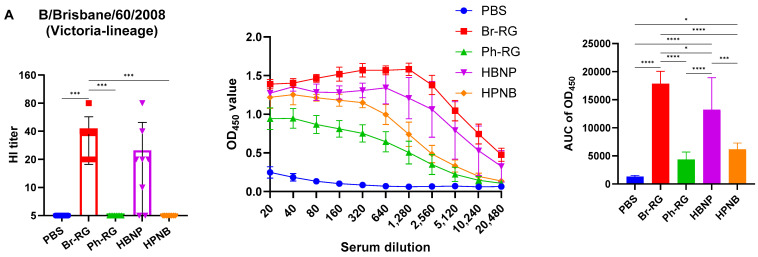

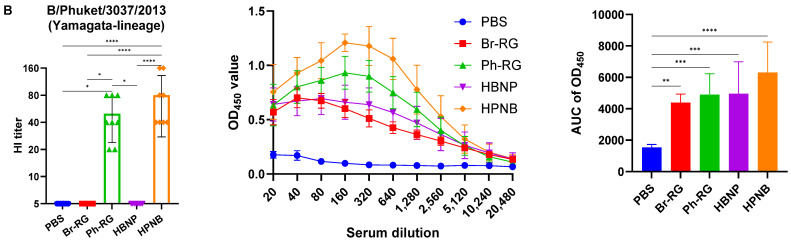
Vaccination with 6 + 1 + 1 constructs induces antibody responses against diverse IBVs. Serum IgG responses to (**A**) Br-wt and (**B**) Ph-wt, as measured by HAI assay and by ELISA. Left panel shows the results of HAI assay (mean ± SD of HAI titers). Middle panel shows the mean ± SD OD_450_ values for serum dilutions in each group in ELISA. Right panel shows the mean + SD of area under the curve (AUC) of OD_450_ values as a readout of ELISA. Data were analyzed by one-way ANOVA with Tukey’s post-hoc multiple analyses test. *—*p* < 0.05; **—*p* < 0.01; ***—*p* < 0.001; ****—*p* < 0.0001.

Interestingly, when the Br-wt virus (B/Victoria lineage) was used as an antigen, sera from animals immunized with homologous HA/NA combination had significantly greater binding signals compared to the group which was vaccinated by the hybrid virus HBNP (Figure 7A), suggesting that the anti-NA binding antibodies were also a significant contributor to the overall immunogenicity. This assumption is supported by the observed stronger binding of the sera from the HPNB group to the Br-wt antigen, compared to the Ph-RG group (Figure 7A).

Notably, IgG responses to the Ph-wt whole virus (Yamagata lineage) in the 6 + 1 + 1 HPNB group were even greater than that of homologous 6 + 2 LAIV Ph-RG group (Figure 7B), most probably due to a higher level of replication of the hybrid vaccine prototype in lung tissues (Figure 6), which could lead to the higher antigenic load and stimulation of a more pronounced humoral immune response. It is also worth noting that the responses induced by both Br-RG and HBNP candidates had a high degree of cross-reactivity to the Ph-wt antigen, with the levels close to that of the Ph-RG vaccine (Figure 7B). These data are in line with previously observed findings on the ability of B/Victoria-lineage type B LAIV, but not B/Yamagata-lineage LAIV virus, to protect ferrets against infections caused by the IBVs of both lineages [36].

Since the hybrid LAIV candidates carried irrelevant NA genes compared to the traditional LAIV reassortants, it was important to measure the levels of induced anti-NA antibodies. Neuraminidase is expressed on the surface of influenza virions and is essential for the release of newly formed virus particles from infected cells [37,38]. An increase in NA inhibition (NAI) titers following vaccination may therefore generate cross-lineage protection. To determine if a two-dose vaccination with 6 + 1 + 1 LAIV vaccines induces anti-NA antibodies, we tested sera from vaccinated mice in an enzyme-linked lectin assay (ELLA). To do this, we rescued two diagnostic Len/17-based influenza A viruses that carry NA ectodomains of Br-wt (H2Br) or Ph-wt (H2Ph) influenza B viruses (Table 1). The use of such viruses in ELLA ensures that there is no interference with antibodies targeting proteins other than NA viral proteins (such as HA or NP).

**Figure 8:**
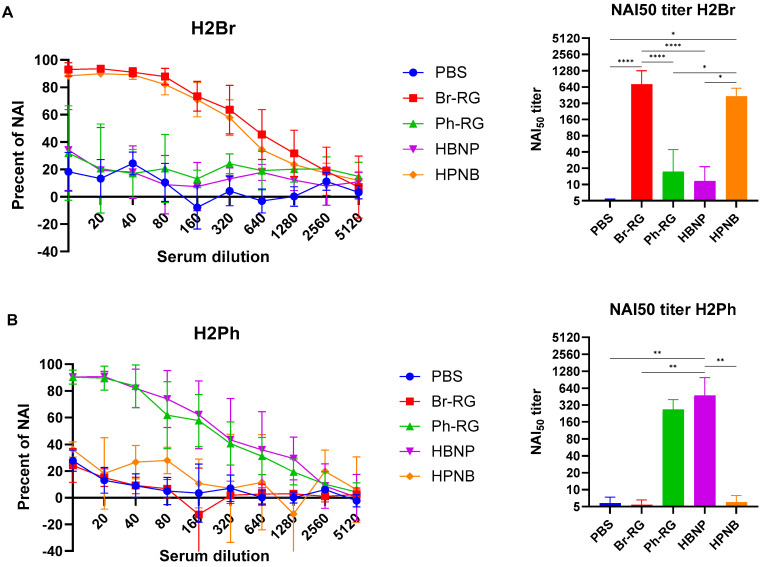
NA-inhibiting (NI) antibody titers in serum. Serial dilutions of 7 + 1 Len/17-based diagnostic strain with NA of Br-wt (**A**) or Ph-wt (**B**) were incubated for 1 h at 37 °C in fetuin-coated plates and the reactivity with PNA-HRPO was determined as described. The dilution of H2Br selected for use in the ELLA was 1:640 and the dilution of H2Ph selected was 1:640 because these dilutions resulted in approximately 90% of the maximum optical density and were within the linear range. Data were compared with one-way ANOVA with Tukey’s post-hoc multiple analyses test. *—*p* < 0.05; **—*p* < 0.01; ****—*p* < 0.0001.

Importantly, all LAIV candidates tested in our study induced high levels of NAI antibodies; however, these antibodies had a very low level of cross-reactivity (Figure 8). Indeed, the Br-NA activity was significantly inhibited by sera from the Br-RG and HPNB vaccine groups, but not from mice immunized with LAIVs that contained Ph-NA (Figure 8A). And vice versa, NAI_50_ titers in sera from animals that received LAIVs expressing NA from the Ph-wt virus were significantly greater than those of the LAIVs carrying NA of the opposite lineage (Figure 8B).

Overall, the hybrid 6 + 1 + 1 LAIV candidates were able to induce high levels of virus-specific antibodies, which were more cross-reactive for the HBNP compared to the HPNB variant. Furthermore, both variants induced substantial levels of NA-inhibiting antibodies which were strongly lineage-specific. These data on the ability of hybrid LAIVs to induce humoral immunity to both IBV lineages suggest that they may have some advantages over classical LAIVs in terms of cross-lineage protection.

### 3.6. Protective Activity of Hybrid IBV LAIV Candidates against Heterologous Influenza B Viruses

**Figure 9:**
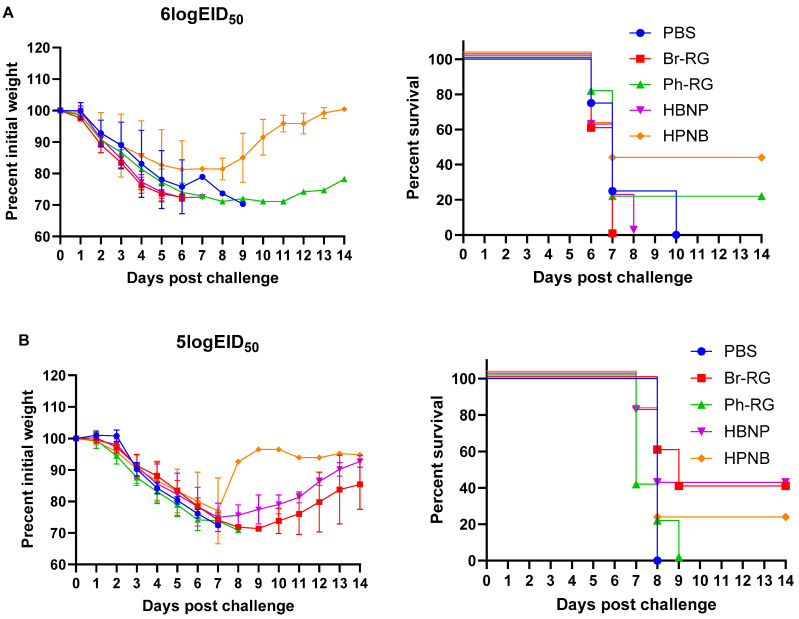
Weight loss and survival of vaccinated groups challenged with (**A**) 6 logEID_50_ or (**B**) 5 logEID_50_ of mouse-adapted B/Malaysia/2506/2004 virus in mice. Left panel shows dynamics of body weight over the challenge phase. Right panel shows survival rates among all mice used in the experiment.

Next, we tested the protective effect of the 6 + 1 + 1 hybrid LAIV candidates against a panel of virulent diverse influenza B viruses. Mice were immunized as described above and were challenged 4 weeks after the last vaccination with IBVs representing the Victoria (Br-wt, Ma-wt) and Yamagata (Ph-wt) lineages. For the mouse-adapted MA-wt challenge, mice were infected i.n. with 6 or 5 logEID_50_ of the virus (corresponded to 30 MLD_50_ and 3 MLD_50_, respectively), and weight loss and survival rates were monitored for 2 weeks after challenge. Most of vaccinated mice succumbed to the infection with either virus dose (Figure 9). When challenged by 30 MLD_50_, mice vaccinated with Ph-RG and HPNB had survival rates of 20% and 40%, respectively (Figure 9A). However, when challenged by 3 MLD_50_ of Ma-wt MA, mice in groups vaccinated by Br-RG and HBNP showed similar 40% survival rates, while in the group vaccinated by HPNB only 20% of mice survived (Figure 9B). There were no significant differences in the dynamics of body weight and survival rate between the study groups. This demonstrated that neither the 6 + 2 nor 6 + 1 + 1 vaccination approach could successfully confer full protection against the heterologous Victoria-lineage mouse-adapted IBVs in mice, and only a moderate level of protection could be achieved.

**Figure 10:**
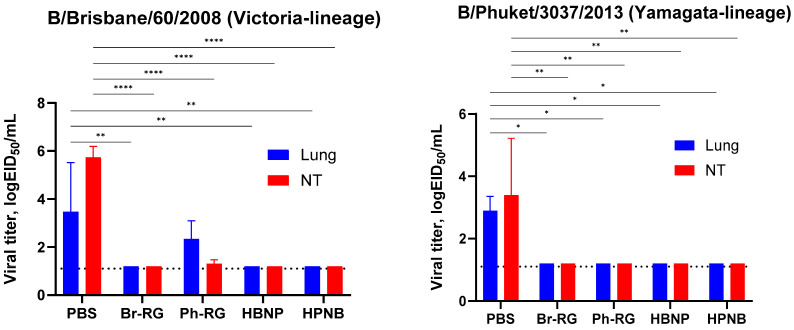
Protective activity of 6 + 2 and 6 + 1 + 1 LAIV viruses in mice. Immunized mice were challenged with B/Brisbane/60/2008 (**left panel**) and B/Phuket/3037/2013 virus (**right panel**), and viral titers were determined in lungs and nasal turbinates on Day 3 post challenge. Data were compared with two-way ANOVA with Tukey’s post-hoc multiple analyses test. *—*p* < 0.05; **—*p* < 0.01; ****—*p* < 0.0001. Dotted line represents the limit of virus detection in the EID_50_ assay.

We further challenged groups of immunized mice with 6 logEID_50_ of either the Br-wt or Ph-wt virus. These viruses were not lethal for mice immunized with corresponding homologous LAIVs, and therefore the protective effect was monitored by the reduction in viral loads in mouse lungs and NTs 3 days after challenge. Similar to the results observed during comparative studies of RG versus non-RG viruses (Figure 4), on Day 3 p.c. almost no infectious titers were found in all vaccine groups. In contrast, the mock group showed relatively high lung and NT virus titers (Figure 10). These data supported our findings that vaccination with 6 + 1 + 1 hybrid type B LAIV candidates and classical 6 + 2 reassortants provides broad cross-protection of mice against a heterologous IBV strain, accompanied by viral clearance in the lungs and NTs. Notably, no significant decrease in viral pulmonary titers was seen for the Ph-RG group challenged with the heterologous Br-wt virus, suggesting incomplete protection (Figure 10). Nevertheless, there were no significant differences in the virological or clinical outcomes of the challenge infection between the 6 + 2 and 6 + 1 + 1 vaccine groups, suggesting that the improvement of the cross-protective potential afforded by the 6 + 1 + 1 LAIV vaccines could not be detected in this experimental model. Most probably, the high cross-protective potential of the traditional LAIVs was mediated by broadly reactive T-cell responses that are mainly targeted at viral internal proteins, such as M and NP. These internal and non-structural proteins of the influenza virus are relatively conserved as a result of sequence functional constraints, and CTL immunity against them is cross-reactive between the two IBV lineages. The internal proteins of the B60 MDV originate from the virus isolated in 1969, but they still contain the CTL epitopes which are conserved in currently circulating IBVs, and vaccination with B60-based LAIVs can trigger T-cell immunity which provides some level of protection against recent viruses [39].

**Figure 11:**
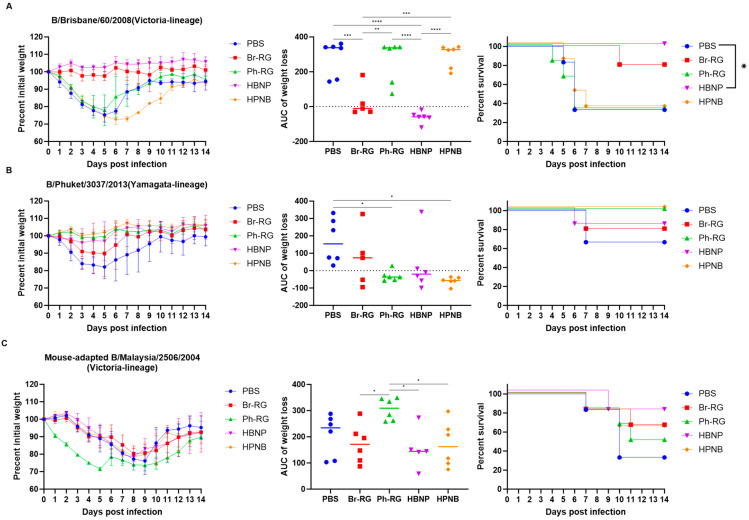
Indirect protective effect of the sera from mice immunized with LAIV prototypes generated in this study. Pooled sera collected from immunized mice at Day 43 were mixed with (**A**) Br-wt, (**B**) Ph-wt, or (**C**) Ma-wt virus at a dose of 3 MLD_50_ and inoculated intranasally to naïve C57BL/6J mice (*n* = 5–6). Survival and weight loss were monitored for 14 days post-challenge. Data were analyzed by one-way ANOVA with Tukey’s post-hoc multiple analyses test. *—*p* < 0.05; **—*p* < 0.01; ***—*p* < 0.001; ****—*p* < 0.0001.

To further confirm that the 6 + 1 + 1 LAIV-induced NA specific antibody can mediate improved cross-protection, without interfering with virus-specific T-cell responses, we assessed the protective effect of immune sera in a mouse in vivo protection model. MLD_50_ values for each challenge virus for C57BL/6J mice were determined in preliminary experiments, and the dose of 3 MLD_50_ of the Br-wt, Ph-wt and Ma-wt viruses was selected for this experiment. Pooled immune sera collected on Day 43 of the experiment were mixed with the viruses at specified doses and i.n. administered to naïve mice (5 or 6 mice per challenge), followed by the observation of weight loss and lethality for two weeks. Interestingly, sera from the HBNP-immunized animals fully protected mice against weight loss and lethality after Br-wt challenge, which was a similar level of protection as afforded by the homologous Br-RG immune sera (Figure 11A). These data confirm that the replacement of Br-NA with Ph-NA in the LAIV reassortant virus does not compromise the protective activity of antibodies against the homologous Br-wt strain. In contrast, neither Ph-RG- nor HPNB-induced antibodies were able to protect mice against Br-wt challenge, suggesting the low cross-reactive potential of these immune sera. 

The challenge of naive mice with Ph-wt could not generate a sufficient level of lethality and there were no significant differences between the groups in terms of survival rates (Figure 11B). Interestingly, the best protection against weight loss was observed in the Ph-RG and HPNB groups, suggesting the most significant impact of HA-specific antibodies. Nevertheless, there was a tendency of better protection in terms of weight loss reduction in the HPNB group compared to the Br-RG, which could be mediated by the NA-inhibiting antibodies.

The protective effect of NA-specific antibodies was clearly seen when immune sera were tested against the B/Malaysia/2506/2004 challenge. This virus belongs to the B/Victoria lineage, and the observation that animals in the HPNB group were less likely to lose weight compared with Ph-RG group suggests that NA can also induce protective antibody against the opposite IBV lineage (Figure 11C). Importantly, despite the differences in the NA content, both the Br-RG and HBNP vaccine candidates demonstrated similarly high levels of protection against the Ma-wt challenge, which was manifested by lower weight loss values and higher survival proportions than in the Ph-RG and the mock groups. 

Overall, based on the results of assessment of safety, immunogenicity and direct and indirect protection studies, the HBNP hybrid variant represents the most promising type B LAIV candidate which is capable of protection against both IBV lineages. This variant was safe in terms of replicative potential in mouse lungs and induced substantial levels of antibodies targeting antigenic determinants of both B/Yamagata and B/Victoria lineage viruses. Finally, several challenge experiments found this variant to have the highest protective levels against diverse influenza B viruses. Given the high growth characteristics of the virus in eggs, it can be easily produced on a manufacturing scale and used as a cross-reactive anti-IBV component of a trivalent seasonal influenza vaccine.

## 4. Discussion

The rescue of recombinant influenza viruses from plasmid DNA is a simple and straightforward process once the protocol is routinely performed in the laboratory [28]. The classical reassortment method which is currently used for the development of reassortant viruses for Russian seasonal LAIV requires 2 to 3 months for vaccine strain generation and characterization. In contrast, only 1–3 weeks would be needed to generate reassortants by the DNA transfection method [40]. The development of these reverse genetics techniques and successful rescue of recombinant influenza viruses from plasmids allows for the more effective production and higher quality of vaccines expressing appropriate HA and NA antigens for seasonal IBV vaccines. In the present study, we report for the first time the successful rescue of B/USSR/60/69-based influenza B LAIV reassortants carrying HA and NA genes of prototype B/Victoria and B/Yamagata lineage viruses. The RG-derived LAIVs demonstrated identical in vitro and in vivo properties as compared to the corresponding LAIV strains generated by classical reassortment in eggs. The development of such an RG system for the B60 master donor virus paved the way for the design of IBV LAIV candidates with improved characteristics.

Although annual seasonal influenza vaccination campaigns contribute significantly to protection against morbidity and mortality, the efficacy of standard licensed vaccines is frequently suboptimal, most often due to mismatches between vaccine strains and circulating viruses [41,42]. For this reason, attempts to develop broad-spectrum influenza vaccines capable of protecting against a broad range of influenza viruses have been ongoing over the past several decades [43,44]. While numerous prototypes exist for influenza A viruses, many of which have already undergone clinical trials [45], the landscape for effective vaccines against influenza B viruses remains comparatively limited [46,47]. A possible reason for the reduced interest in developing cross-protective vaccines against influenza B viruses is the less severe course of the disease and slower rate of virus evolution compared to influenza A viruses, as well as the licensing of quadrivalent vaccines that include both IBV lineages and thus eliminate the possibility of incorrectly predicting which lineage would circulate in the next season [26,48]. Nevertheless, vaccine effectiveness may still be reduced in the case of antigenic drift within either IBV lineage [49]. Moreover, in the case of licensed live attenuated vaccines, the addition of a fourth viral component may have a negative effect on vaccine effectiveness probably due to the interference between the IBV and IAV strains [27]. Therefore, in our study, we sought to develop a cross-lineage type B LAIV that was capable of generating cross-reactive antibodies against both B/Yamagata and B/Victoria lineages, and whose inclusion into trivalent LAIV would provide broad cross-protection against various IBVs.

Most seasonal vaccines predominantly trigger the production of neutralizing antibodies, focusing on the hypervariable epitopes of the HA head domain, resulting in a limited range of protection [50,51]. Epitopes in the HA stalk have a broader cross-reactive potential, and therefore several studies have been focusing on improving the broadly protective response against the HA stalk domain [52,53,54,55]. IBV infection and immunization induce antibodies specific to the HA head and stalk, but only HA stalk-specific antibodies display cross-reactivity with IBV of both lineages [56,57].

Another major surface glycoprotein, neuraminidase, undergoes antigenic drift as well, similar to HA. However, this process is slower and acts independently of the antigenic drift observed in HA [58,59]. The NA protein is crucial for cleaving terminal sialic acid residues present on host glycoproteins. This cleavage is essential for the release of newly generated virus particles [60,61]. Early studies have demonstrated that antibodies specifically targeting NA are protective, effectively reducing virus shedding and mitigating the severity of infections [62]. Studies in mice have shown that antibodies inhibiting NA are associated with a reduction in lung viral titers [63]. Therefore, NA stands out as a promising target for the development of universal influenza vaccines, offering the potential for broader and more enduring protection against diverse influenza strains [64,65].

In the current study, we explored the possibility of combining protective effects of anti-HA and anti-NA antibodies by designing hybrid influenza B viruses prepared on the B/USSR/60/69 LAIV backbone. In this case, HA and NA genes come from the opposite IBV lineages, thus increasing the repertoire of the induced virus-specific antibodies. We used prototype B/Brisbane/60/2008 (B/Victoria lineage) and B/Phuket/3037/2013 (B/Yamagata lineage) viruses to rescue two hybrid LAIV strains using the established RG system for the B/60 master donor virus. The HBNP strain contains the HA of the Br-wt virus and NA of the Ph-wt virus. In contrast, the HPNB variant expressed the HA of the Ph-wt and NA of the Br-wt strain. Interestingly, such reassorting of the HA and NA genes had no effect on the viral growth characteristics in vitro, but both hybrid viruses were more likely to replicate in mouse lungs compared to classical 6 + 2 LAIV reassortants. This phenomenon will need to be studied in more detail to understand how such HA/NA combinations might have affected the fitness of the virus for lung tissue.

Importantly, the intranasal immunization of mice with the new hybrid LAIV prototypes led to the induction of cross-reactive serum IgG antibodies that were capable of binding with both B/Victoria- and B/Yamagata-lineage viruses. Notably, the LAIVs with HAs originating from the Br-wt virus were more cross-reactive than that containing the HA of the Ph-wt virus, a phenomenon previously observed in a ferret study of monovalent type B LAIVs [36]. Another important observation in this study is that all LAIV prototypes induced robust NA-inhibiting antibodies after primed-boost i.n. immunization. Surprisingly, these antibodies had very low levels of cross-reactivity, further emphasizing the need to develop more broadly reactive antigens as a target for a universal vaccine, since the use of the NA protein from one lineage may have a low protective effect against IBV from the opposite lineage. Nevertheless, given that the hybrid LAIV prototypes induced cross-reactive antibodies in ELISA at levels similar to the corresponding classical LAIVs carrying the same HA, the addition of the NA antigen from the opposite lineage is favorable in terms of an overall broader antibody response compared to standard LAIV strains. Indeed, the LAIV HBNP hybrid variant showed the highest level of cross-reactivity of induced immune responses among all candidates tested, and this immunity showed the best protective potential against various influenza B viruses in both direct and indirect protection studies in mice. An important limitation of our study is that we did not measure the mucosal immune responses to the newly generated viruses, as it is known that IgA antibodies are a major correlate of protection afforded by live virus vaccines [66,67]. Nevertheless, the fact that our hybrid vaccines retained all the properties inherent in classical LAIVs suggests that similar levels of mucosal IgA antibodies would be induced by intranasal immunization. We also did not follow the longevity of the antibody responses to the newly constructed LAIV hybrid prototypes. Traditional LAIVs were shown to induce B- and T-cell responses that persist for at least one year after vaccination [68], and it will be important to demonstrate in future studies whether both the HA- and NA-specific antibody responses are long-lived after immunization with our hybrid IBV LAIV reassortants.

It is also worth noting that LAIVs are efficient inducers of T-cell immunity, which represents another mode of protection against heterologous influenza viruses [39]. It is also known that after influenza B virus infections, cross-reactive CD8+ cytotoxic T-lymphocytes can also be induced [69,70] and may confer a degree of protection against subsequent infections with antigenically distinct influenza B viruses, where antibodies induced by previous infections might not be fully protective [71]. Therefore, the newly generated hybrid LAIV candidates will not only induce broadly reactive HA- and NA-specific antibodies to both lineages of influenza B virus, but also generate long-lived memory T cells to conserved epitopes of internal virus proteins.

## 5. Conclusions

The urgent need for broadly protective influenza B virus vaccines is underscored by the significant burden of IBV in the human population and the relatively high mutation rate of these viruses. The present study focuses on the development of a reverse genetics system for the cold-adapted B/USSR/60/69 master donor virus and the design of novel prototypes of cross-lineage IBV LAIVs with a 6 + 1 + 1 genome composition based on the B60 backbone. The B60 RG system was successfully developed and used for the rescue of the hybrid LAIV candidates with HA and NA genes belonging to the opposite IBV lineages. These 6 + 1 + 1 vaccine viruses maintained comparable growth properties and were shown to generate high levels of cross-reactive HA- and NA-specific antibodies, and these antibodies conferred protection against diverse influenza B viruses. While these results provide valuable insights into the development of broadly protective recombinant influenza virus vaccines, further studies are warranted to confirm their safety, immunogenicity and cross-protective potential using more relevant animal models, such as ferrets. Additionally, the assessment of cross-reactive cell-mediated immune responses generated by vaccination emerges as a crucial factor influencing the ability to protect against both homologous and heterologous challenge viruses. This multifaceted approach to vaccine development, combining genetic engineering with immune response considerations, represents a significant step towards addressing the challenges posed by the dynamic nature of influenza B viruses.

## Figures and Tables

**Table 1:** List of influenza viruses used in this study.

Virus Designation	HA Gene	NA Gene	Six Remaining Segments	Virus Description
**Br-wt**	Br-wt	Br-wt	Br-wt	Wild-type B/Brisbane/60/2008 (B/Victoria lineage)
**Ph-wt**	Ph-wt	Ph-wt	Ph-wt	Wild-type B/Phuket/3037/2013 (B/Yamagata lineage)
**Br-vac**	Br-wt	Br-wt	B60	B/60/Brisbane/60/2008 LAIV reassortant
**Ph-vac**	Ph-wt	Ph-wt	B60	B/60/Phuket/3037/2013 LAIV reassortant
**Br-RG**	Br-wt	Br-wt	B60	RG copy of LAIV based on B/Brisbane/60/2008
**Ph-RG**	Ph-wt	Ph-wt	B60	RG copy of LAIV based on B/Phuket/3037/2013
**B60**	B60	B60	B60	B/USSR/60/69 master donor virus
**B60-RG**	B60	B60	B60	RG copy of B/USSR/60/69 master donor virus
**Ma-wt**	Ma-wt	Ma-wt	Ma-wt	Wild-type B/Malaysia/2506/2004 mouse-adapted virus
**Lee-wt**	Lee-wt	Lee-wt	Lee-wt	Wild-type B/Lee/1940 virus
**HBNP**	Br-wt	Ph-wt	B60	Hybrid LAIV strain with HA of Br-wt and NA of Ph-wt
**HPNB**	Ph-wt	Br-wt	B60	Hybrid LAIV strain with HA of Ph-wt and NA of Br-wt
**H2Br**	Len/17	Br-wt	Len/17	Len/17-based diagnostic strain with NA of Br-wt
**H2Ph**	Len/17	Ph-wt	Len/17	Len/17-based diagnostic strain with NA of Ph-wt

Len/17: A/Leningrad/134/17/57 (H2N2) master donor virus for type A LAIVs.

**Table 2:** Growth characteristics of the studied viruses at different temperatures.

Virus	Viral Titer in Eggs at Indicated Temperature, logEID_50_/mL ± SD				Viral Titer in MDCK Cells, logTCID_50_/mL ± SD	Phenotype
	26 °C	33 °C	37 °C	38 °C		
B60	8.0 ± 0.4	9.7 ± 0.7	3.3 ± 0.9	1.4 ± 0.2	8.1 ± 0.2	*ts/ca*
B60-RG	6.5 ± 0.5	8.4 ± 0.7	2.3 ± 0.4	1.2 ± 0.0	7.8 ± 0.4	*ts/ca*
Br-vac	5.9 ± 0.6	8.4 ± 0.2	1.2 ± 0.0	1.2 ± 0.0	8.5 ± 0.5	*ts/ca*
Br-RG	5.8 ± 0.4	8.5 ± 0.5	1.7 ± 0.9	1.2 ± 0.0	7.9 ± 0.3	*ts/ca*
Br-wt	3.3 ± 0.2	8.6 ± 0.9	1.2 ± 0.0	1.2 ± 0.0	7.8 ± 0.2	*ts/non ca*
Ph-vac	7.9 ± 1.4	8.6 ± 0.6	3.4 ± 0.9	2.4 ± 0.2	8.1 ± 0.5	*ts/ca*
Ph-RG	7.9 ± 0.4	9.5 ± 0.4	4.1 ± 0.1	2.4 ± 0.9	8.5 ± 0.4	*ts/ca*
Ph-wt	7.1 ± 0.3	9.1 ± 0.4	2.0 ± 1.1	1.2 ± 0.0	8.2 ± 0.2	*ts/ca*

*ts*: temperature-sensitive; *ca*: cold-adapted; *non ca*: non-cold-adapted.

**Table 3:** Growth characteristics of engineered influenza B viruses at different temperatures.

Virus	Viral Titer in Eggs at Indicated Temperature, logEID_50_/mL ± SD				Viral Titer in MDCK Cells, logTCID_50_/mL ± SD	Phenotype
	26 °C	33 °C	37 °C	38 °C		
B60	8.0 ± 0.4	9.7 ± 0.7	3.3 ± 0.9	1.4 ± 0.2	8.1 ± 0.2	*ts/ca*
B60-RG	6.5 ± 0.5	8.4 ± 0.7	2.3 ± 0.4	1.2 ± 0.0	7.8 ± 0.4	*ts/ca*
Br-RG	5.8 ± 0.4	8.5 ± 0.5	1.7 ± 0.9	1.2 ± 0.0	7.9 ± 0.3	*ts/ca*
HBNP	6.4 ± 0.8	8.9 ± 0.6	3.3 ± 1.2	1.7 ± 0.5	7.0 ± 0.3	*ts/ca*
Br-wt	3.3 ± 0.2	8.6 ± 0.9	1.2 ± 0.0	1.2 ± 0.0	7.8 ± 0.2	*ts/non ca*
Ph-RG	7.9 ± 0.4	9.5 ± 0.4	4.1 ± 0.1	2.4 ± 0.9	8.5 ± 0.4	*ts/ca*
HPNB	7.2 ± 0.6	9.3 ± 0.5	2.6 ± 1.0	2.0 ± 0.5	7.3 ± 0.3	*ts/ca*
Ph-wt	7.1 ± 0.3	9.1 ± 0.4	2.0 ± 1.1	1.2 ± 0.0	8.2 ± 0.2	*ts/ca*

*ts*: temperature-sensitive; *ca:* cold-adapted; *non ca*: non-cold-adapted.

## Data Availability

The data presented in this study are available on request from the corresponding author.

## Supplementary Materials

The following supporting information can be downloaded at: https://www.mdpi.com/article/10.3390/vaccines12010095/s1, Table S1: Primer set used for RT-PCR amplification of the eight vRNAs of B/USSR60/69, HA and NA genes of B/Brisbane/60/2008 and B/Phuket/3037/2013; NA genes of 7 + 1 A/H2NBr and A/H2NPh. Figure S1: RG plasmids carrying six internal protein segments of B/USSR/60/69 (B60) master donor virus, non-treated (NT) or hydrolyzed (H) with XbaI restriction enzyme.
