# Supplementary material for: Development of Cross-Reactive Live Attenuated Influenza Vaccine Candidates against Both Lineages of Influenza B Virus
Source: Vaccines (Basel). 2024 Jan 18;12(1):95. doi: 10.3390/vaccines12010095 (PMC10821225; doi:10.3390/vaccines12010095)
Supplement: Supplementary file 1 [file vaccines-12-00095-s001.zip › vaccines-2784063-supplementary.pdf]

Supplementary Materials

Table S1. Primer set used for RT-PCR amplification of the eight vRNAs of B/USSR60/69, HA and NA genes of B/Brisbane/60/2008 and B/Phuket/3037/2013; NA genes of 7+1 A/H2NBr and A/H2NPh.

Primer name	5'-Sequence-3' (*)
B-Uni_PB2-SapI_F	GATCGCTCTTCAGGG **AGCAGAAGCGGAGCGTTTTCAAGATG**
B-Uni_PB2-SapI_R	ACTGGCTCTTCTATT **AGTAGAAACACGAGCATT**
B-Uni_PB1-SapI_F	GATCGCTCTTCAGGG **AGCAGAAGCGGAGCCTTTAAGATG**
B-Uni_PB1-SapI_R	ACTGGCTCTTCTATT **AGTAGAAACACGAGCCTT**
B-Uni_PA-SapI_F	GATCGCTCTTCAGGG **AGCAGAAGCGGTGCGTTTGA**
B/USSR_PA-BsmBI-1261R	TATTCGTCTCC **CAGGGCCCTTTTACTTGTCAGAGTAC**
B/USSR_PA-BsmBI-1283F	TATTCGTCTCT **CCTGGATCTACCAGAAATAGGGCCAGAC**
B-Uni_PA-SapI_R	ACTGGCTCTTCTATT **AGTAGAAACACGTGCATT**
B-Uni_HA-SapI_F	GATCGCTCTTCAGGG **AGCAGAAGCAGAGCATTTTCTAATATC**
B-Uni_HA-SapI_R	ACTGGCTCTTCTATT **AGTAGTAACAAGAGCATTTTTC**
B-Uni_NP-SapI_F	GATCGCTCTTCAGGG **AGCAGAAGCACAGCATTTTCTTGTG**
B/USSR-NP-1411R	**ACCCTCCGTCTCCACCTACTTCA**
B/USSR-NP-1433F	**TGAAGTAGGTGGAGACGGAGGGT**
B-Uni_NP-SapI_R	ACTGGCTCTTCTATT **AGTAGAAACAACAGCATTTTTTAC**
B-Uni_NA-SapI_F	GATCGCTCTTCAGGG **AGCAGAAGCAGAGCA**
B-Uni_NA-SapI_R	ACTGGCTCTTCTATT **AGTAGTAACAAGAGCATTTT**
B-Uni_M-SapI_F	GATCGCTCTTCAGGG **AGCAGAAGCACGCACTTTCTTAAAATG**
B-Uni_M-SapI_R	ACTGGCTCTTCTATT **AGTAGAAACAACGCACTTTTTCCAG**
B-Uni_NS-SapI_F	GATCGCTCTTCAGGG **AGCAGAAGCAGAGGATTTGTTTAGTC**
B-Uni_NS-SapI_R	ACTGGCTCTTCTATT **AGTAGTAACAAGAGGATTTTTAT**
NA_B_H2_F	**AGCAAAAGCAGGAGTGAAAATGCTACCTTCAACTATAC**
NA_B_Bris_H2_H3_R	**AGTAGAAACAAGGAGTTTTTTCTAAAATTGCGAAAGCTTACAGAGCCATGTC**
NA_B_Phu_H2_H3_R	**AGTAGAAACAAGGAGTTTTTTCTAAAATTGCGAAAGCTTACAGAGTCATATTAACAC**

(*) The sequences complementary to the influenza sequences are shown in bold. The 5′-ends have recognition sequences for the restriction endonuclease SapI or BsmBI. The design of the primers for PA and NP allowed the amplification of two fragments.

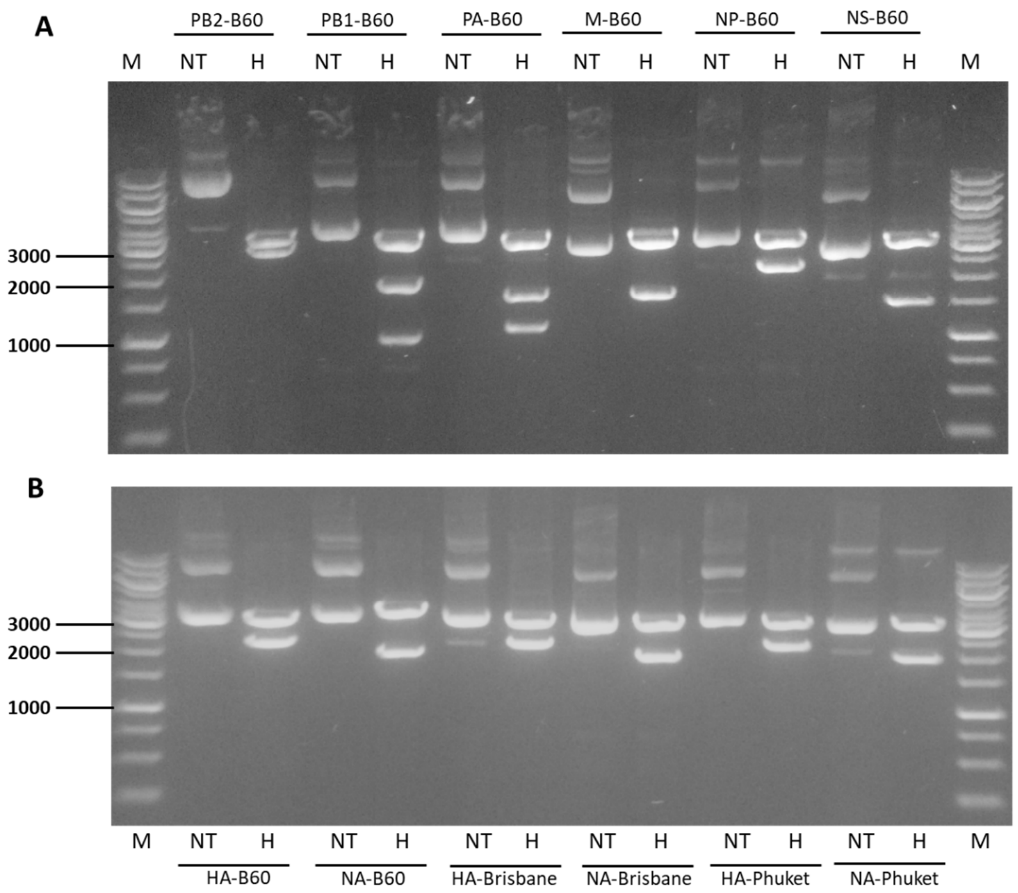

Figure S1. (A). RG plasmids carrying six internal protein segments of B/USSR/60/69 (B60) master donor virus, non-treated (NT) or hydrolyzed (H) with XbaI restriction enzyme. **(B).** RG plasmids carrying HA or NA segments of B/USSR/60/69 (B60) master donor virus or B/Brisbane/60/2008 or B/Phuket/3037/2013 wild-type viruses, non-treated (NT) or hydrolyzed (H) with XbaI restriction enzyme. M- DNA ladder.
